# Diversity of Tumor-Infiltrating, γδ T-Cell Abundance in Solid Cancers

**DOI:** 10.3390/cells9061537

**Published:** 2020-06-24

**Authors:** Ghita Chabab, Florence Boissière-Michot, Caroline Mollevi, Jeanne Ramos, Evelyne Lopez-Crapez, Pierre-Emmanuel Colombo, William Jacot, Nathalie Bonnefoy, Virginie Lafont

**Affiliations:** 1 IRCM, Institut de Recherche en Cancérologie de Montpellier, INSERM U1194, Université de Montpellier, Institut régional du Cancer de Montpellier, 34298 Montpellier, France; ghita.chabab@inserm.fr (G.C.); Evelyne.Crapez@icm.unicancer.fr (E.L.-C.); Pierre-Emmanuel.Colombo@icm.unicancer.fr (P.-E.C.); william.jacot@icm.unicancer.fr (W.J.); nathalie.bonnefoy@inserm.fr (N.B.); 2 Translational Research Department, Institut régional du Cancer de Montpellier, 34298 Montpellier, France; Florence.Boissiere@icm.unicancer.fr (F.B.-M.); Jeanne.Ramos@icm.unicancer.fr (J.R.); 3 Institut régional du Cancer de Montpellier, 34298 Montpellier, France; Caroline.Mollevi@icm.unicancer.fr

**Keywords:** γδ T-cell, cancer, tumor microenvironment, immune infiltrate, immunohistochemistry, TNM staging

## Abstract

γδ T-cells contribute to the immune response against many tumor types through their direct cytolytic functions and their capacity to recruit and regulate the biological functions of other immune cells. As potent effectors of the anti-tumor immune response, they are considered an attractive therapeutic target for immunotherapies, but their presence and abundance in the tumor microenvironment are not routinely assessed in patients with cancer. Here, we validated an antibody for immunohistochemistry analysis that specifically detects all γδ T-cell subpopulations in healthy tissues and in the microenvironment of different cancer types. Tissue microarray analysis of breast, colon, ovarian, and pancreatic tumors showed that γδ T-cell density varies among cancer types. Moreover, the abundance of γδ tumor-infiltrating lymphocytes was variably associated with the outcome depending on the cancer type, suggesting that γδ T-cell recruitment is influenced by the context. These findings also suggest that γδ T-cell detection and analysis might represent a new and interesting diagnostic or prognostic marker.

## 1. Introduction

The malignant features of cancer cells are tightly regulated by their local environment and by the network they form with host cells, such as immune cells, angiogenic vascular cells, endothelial cells, and cancer-associated fibroblasts (cancer ecosystem). The ability of tumor cells to overcome immune surveillance is an essential step in tumor progression. In this context, the nature and frequency of tumor infiltrating immune cells, particularly cytotoxic CD8 T-cells, are considered to be prognostically significant in many cancer types. Indeed, stronger CD8 T-cell infiltration in the tumor is generally associated with better prognosis. This has been demonstrated in various cancers, such as melanoma [1], ovarian [2], colorectal [3], bladder [4], breast [5], and pancreatic cancer [6]. Besides CD8 T-cells, other immune cell types are involved in tumor control or progression as regulatory T-cells (Treg), macrophages, and γδ T-cells.

γδ T-cells are non-conventional T lymphocytes, with a T-cell receptor (TCR) composed of a γ and a δ chain. Depending on the TCR structure, human γδ T-cells can be divided in three main subpopulations: Vδ1, Vδ2, and Vδ3 T-cells [7,8]. Vδ2 T-cells are the main subtype (90%) of γδ T-cells in peripheral blood. The Vδ1 and Vδ3 subsets are mostly found in tissues and mucosa, respectively. γδ T-cells are involved in the immune response against many cancers (e.g., myeloma, melanoma, breast, colon, lung, ovary, and prostate), and can be found in the tumor microenvironment (TME) [9,10,11,12,13]. Their anti-tumor effect relies on their direct cytolytic activity against transformed cells and their ability to stimulate and regulate the biological functions of other cell types, such as dendritic cells (DC), interferon-γ-producing CD8 αβ T-cells, and natural killer (NK) cells, that are required for the initiation and the establishment of an efficient anti-tumor immune response [14,15,16,17]. Unlike conventional αβ T-cells, γδ T-cells display a potent MHC-independent reactivity against a broad panel of tumors. They also show limited, if any, alloreactivity, and can be massively and specifically expanded from samples (e.g., peripheral blood). For these reasons, γδ T-cells are considered highly attractive therapeutic targets for anti-tumor immunotherapies. Although human Vδ1, Vδ2, and Vδ3 T-cells show a strong reactivity against tumor cells, γδ T-cell-based immunotherapies primarily target the Vδ2 subset, because they can be easily expanded and specifically activated with synthetic clinical-grade phosphoantigens (e.g., BrHPP), or with pharmacological inhibitors of the mevalonate pathway (e.g., zoledronate), which produces physiologic phosphoantigens [18,19]. Although showing interesting results, 70% to 90% of patients did not respond to the treatment, even if patients exhibited a significant Vδ2 proliferation and activation. This suggested that either immunosuppressive mechanisms present in the TME inhibit Vδ2 anti-tumor functions, or that other γδ subtypes are involved in the anti-tumor response, such as Vδ1 T-cells. Vδ1 T-cells are usually predominant (compared with Vδ2) in solid tumor infiltrates, and react efficiently against tumor cells by displaying potent cytotoxic activities [10,20]. Another hypothesis is that immunosuppressive γδ T-cells are also expanded. In line with these hypotheses, several reports have brought evidence that γδ T-cells can be associated with pro-tumor functions in some cancers, or in contrast, limit tumor progression. Regarding these “conflicting” statements, a large study of γδ T-cell infiltrate in various cancers has to be done, in order to decipher their implication in cancer development and be able to declare γδ T-cells as a prognosis marker.

To date, the detection of tumor-infiltrating γδ T-cells remains a challenge. Indeed, although several studies have previously evaluated γδ T-cell density analysis in cancer, the detection of tumor infiltrating γδ T-cells by immunohistochemistry (IHC) has been hampered by their unreliable recognition by antibodies, or the unavailability of antibodies leading to non-reproducible intra- and inter-laboratory results. 

Here, we validated an anti-TCRδ antibody that allows γδ T-cell detection by IHC in formalin-fixed paraffin-embedded (FFPE) tissues. We analyzed the frequency of γδ T-cells in tissue microarrays (TMAs) of breast, colon, ovarian, and pancreatic cancers, and of representative normal tissue samples. We found that γδ T-cell density varied among cancer types. Moreover, γδ tumor-infiltrating lymphocyte (TIL) frequency was variably associated with tumor stage (tumor, node, and metastasis; TNM), suggesting that distinct contexts underlie γδ TIL recruitment, or that pro-tumor γδ TIL subsets are specifically recruited or differentiate in the tumor microenvironment.

## 2. Materials and Methods

### 2.1. Sample Collection

Tissue samples were selected from the Montpellier Cancer Institute (ICM) biological resources center. Clinical data (e.g., age, treatment, TNM, grade) were obtained by reviewing the medical files. Samples were collected following French laws under the supervision of an investigator, and their collection was declared to the French Ministry of Higher Education and Research (declaration number DC-2008–695). The study was approved by the Montpellier Cancer Institute Institutional Review Board (ICM-CORT-2015-32, ICM-CORT-2019-06, and ICM 2019-12). A total of 418 samples were included in this retrospective study.

### 2.2. Breast Cancer Tissue Microarray

A tissue microarray (TMA) with breast tumor samples from 50 chemotherapy-naïve patients was constructed using two malignant tissue cores (1 mm diameter) per tumor. Healthy breast tissues were from another breast TMA, because no healthy tissue sample was collected for this group of patients. The main clinicopathological characteristics of this cohort are presented in Table S1.

### 2.3. Colorectal Cancer Tissue Microarray

Two TMAs were used for colorectal cancer investigation. The first TMA included 59 colorectal tumors (24 primary tumors and 35 metastatic tumors), and was constructed using two malignant tissue cores (1 mm diameter) per tumor. The second TMA included 53 samples from 46 patients, and was constructed using two malignant tissue cores (1 mm diameter) per tumor, and matched normal mucosa (distal or proximal) when available. The main clinicopathological characteristics of this cohort are presented in Table S2.

### 2.4. Pancreatic Cancer Tumor Microarray

A TMA of pancreatic cancer specimens from 50 patients was constructed using two malignant tissue cores (1 mm diameter) per tumor, and matched normal tissue and epineoplastic pancreatitis when available. The main clinicopathological characteristics of this cohort are presented in Table S3.

### 2.5. Ovarian Cancer Tumor Microarray

A TMA of ovarian cancer specimens from 72 patients was constructed using two malignant tissue cores (1 mm diameter) per tumor, associated with one matched normal tissue core when available. The main clinicopathological characteristics of this cohort are presented in Table S4.

### 2.6. Immunohistochemistry

For each TMA, 3 µm thin sections were mounted on Flex microscope slides (Agilent, Glostrup, Denmark) and dried overnight at room temperature before IHC. The PTLink system (Agilent, Glostrup, Denmark) was used for de-paraffinization and heat-induced antigen retrieval in High pH Buffer (Agilent, Glostrup, Denmark) at 95 °C for 15 min. IHC was performed using the Dako Autostainer Link48 platform (Agilent, Glostrup, Denmark). Briefly, endogenous peroxidase was quenched by incubation with Flex Peroxidase Block (Agilent, Glostrup, Denmark) at room temperature for 5 min. Then, slides were incubated with the anti-TCRδ mouse monoclonal antibody (Clone H-41, Santa Cruz) (1/150 dilution) at room temperature for 30 min. Signal amplification was performed using a mouse linker (Agilent, Glostrup, Denmark) for 15 min, followed by two rinses in EnVision FLEX Wash buffer (Agilent, Glostrup, Denmark), and then incubation with a horseradish peroxidase-labeled polymer coupled to secondary anti-mouse and anti-rabbit antibodies for 20 min. During this process, 3,30-Diaminobenzidine was used as substrate. Sections were counterstained with Flex Hematoxylin (Agilent, Glostrup, Denmark), followed by rinsing in tap water for 5 min. Finally, sections were dehydrated and mounted with a coverslip.

The NanoZoomer slide scanner system (Hamamatsu Photonics) and a ×20 objective were used to digitalize the TMAs after staining. Immunoreactive cells were manually identified and counted on the digitalized slides with NDP.view software. When both cores from the same tumor were assessable, the mean value was calculated and used for statistical analysis, and the data expressed as the number of TCR γδ-positive cells per mm^2^. The mean and SEM were then calculated by using the mean value of the two assessable cores when possible, and the raw density (value of the single spot) for tumor samples with only one assessable core. For healthy tissue samples, only one core was available per patient.

### 2.7. In Situ Hybridization

RNA in situ hybridization of TMA sections was performed using specific human TCRD probes and the RNAscope 2.5 HD detection kit, according to the manufacturer’s instructions (ACD, Newark, CA, USA). In brief, 5 μm thin sections were deparaffinized and placed in xylene, followed by absolute ethanol. Slides were then pretreated with a pretreatment reagent kit (ACD, Newark, CA, USA) for 15 min at 100 °C, according to the manufacturer’s instructions. Following a protease step to permeabilize tissue (15 min at 40 °C), slides were then hybridized with the specific probes (Advanced Cell Diagnostics, Hayward, CA, USA) at 40 °C for 2 h in a HybEZ hybridization oven, and amplified sequentially following the 2.0 HD detection kit–brown (ACD, Newark, CA, USA) per the manufacturer’s instructions. Besides the specific TCRD probe, probes that target the human housekeeping *PPIB* gene and the bacterial *DapB* gene were used as positive and negative controls, respectively.

### 2.8. TIL Infiltration Assessment

Hematoxylin and eosin-stained (HES) slides were scored for stromal TILs by a senior pathologist. Inflammatory infiltrate was evaluated only in TMA samples with invasive tumors. Inflammatory infiltrates in the stroma of noninvasive lesions and normal structures were excluded. For breast cancer, guidelines for TIL infiltration scoring advocated for clinical management were followed [21]. For colorectal, pancreatic, and ovarian samples, the pathologist first assessed the amount of stroma present on each sample (%*S*). Then, the percentage of tumor stroma area that was occupied by mononuclear inflammatory cells was evaluated (%*M*). The TIL infiltration score was defined as:

(1) $$\frac{\%S\times \%M}{100}$$

### 2.9. Statistical Analysis

Data are presented as scatter plots showing the mean values with the standard error of the mean (SEM). Results were compared using either a Mann–Whitney *t*-test (when two datasets were compared) or one-way ANOVA, Kruskal–Wallis, and Dunn’s multiple comparison tests (when three or more datasets were compared), depending on the experiment. Association analyses were performed using Pearson’s chi-squared test. A value of *p* < 0.05 was considered statistically significant. Analyses were performed using GraphPad Prism, version 6 (San Diego, CA, USA).

## 3. Results

### 3.1. γδ T-Cell Staining by Immunohistochemistry 

**Figure 1:**
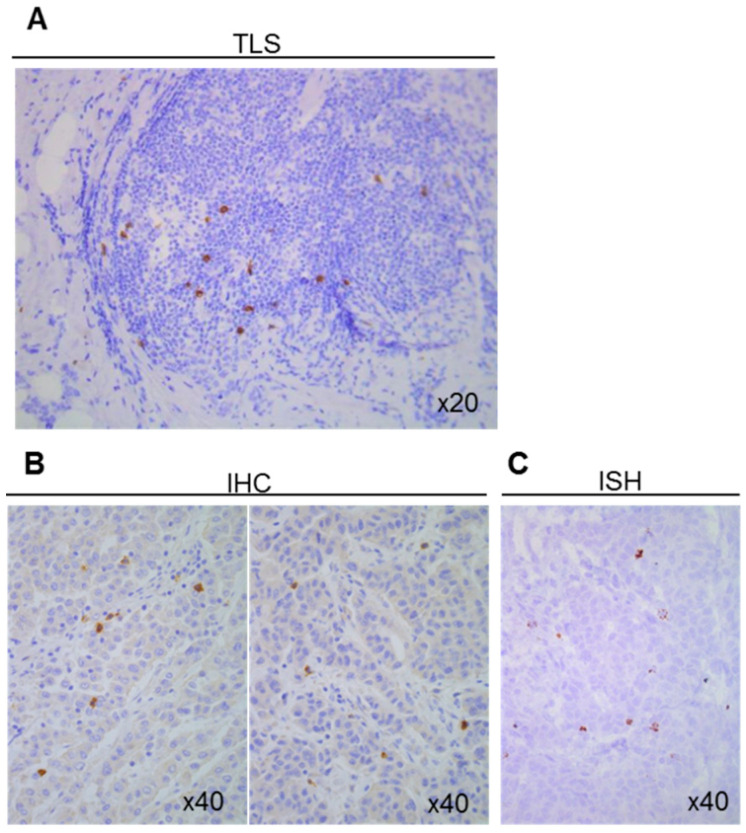
Detection of γδ T-cells using the H-41 antibody. (**A**) Detection of γδ T-cells by immunohistochemistry in a tertiary lymphoid structure (TLS) located close to a breast tumor. Detection of γδ T-cells in colon cancer sections by (**B**) immunohistochemistry (IHC) and (**C**) in situ hybridization (ISH).

To evaluate the ability of the anti-TCR*δ* monoclonal antibody H-41 to detect γδ T-cell populations, we used cell suspensions composed of γδ T-cell-depleted PBMCs with 0%, 50%, and 100% of purified γδ T-cells. Cell pellets were embedded in an aqueous gel solution to test the H-41 antibody. The H-41 antibody detected γδ T-cells, and enabled their precise quantification (0%, 50% or 100%) (Figure S1). The staining of a tertiary lymphoid structure from a patient with breast cancer confirmed that the H-41 antibody can detect γδ T-cells in structures where γδ T-cells are supposed to be found (Figure 1A). To confirm the antibody specificity, we compared γδ T-cell detection by IHC and in situ hybridization in two adjacent colon cancer tissue sections. The pattern of γδ T-cells detected by the two techniques was comparable (Figure 1B–C).

These data demonstrate that the H-41 anti-TCRδ antibody is a robust tool for the detection and quantification of γδ T-cells in FFPE samples by IHC.

### 3.2. Presence of γδ T Cells in Healthy Tissues

**Figure 2:**
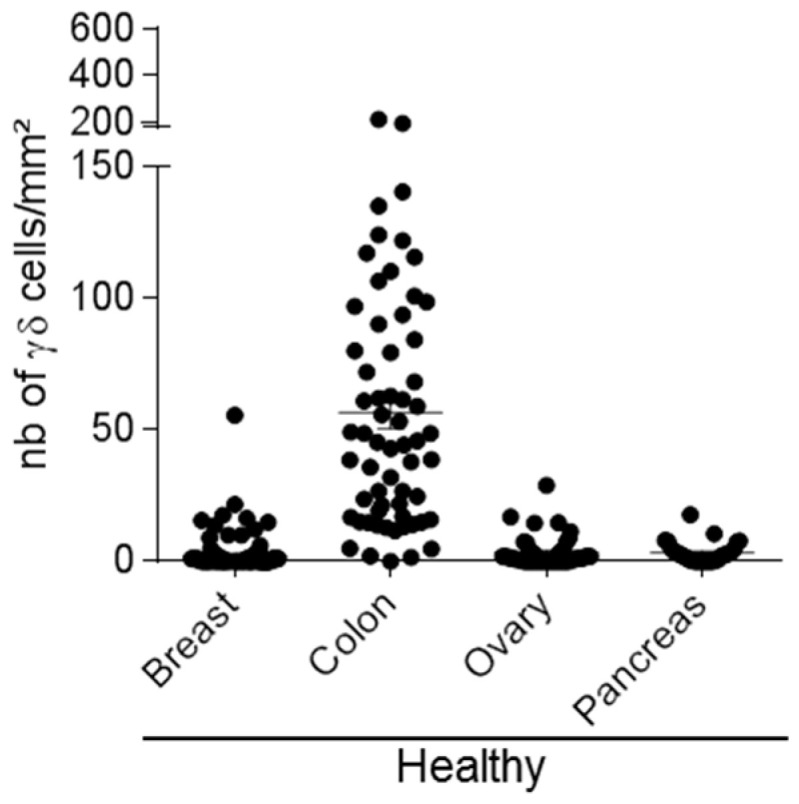
Heterogeneity of γδ T-cell density in normal tissues. Scatter plot showing γδ T-cell density assessed by IHC in tissue microarrays (TMAs) with normal breast (*n* = 141), colon (*n* = 62), ovary (*n* = 49), and pancreas (*n* = 31) samples. Data are presented as the mean ± SEM.

We first investigated the presence of γδ T-cells in sections from healthy colon (*n* = 62), ovary (*n* = 49), breast (*n* = 141), and pancreas (*n* = 31) samples. We observed a great heterogeneity. Indeed, γδ T-cells were abundant in normal colon (1 to 213 cells/mm^2^) and in some breast tissue samples (0 to 55 cells/mm^2^). Conversely, we detected only few γδ T-cells in normal pancreatic (0 to 17 cells/mm^2^) and ovarian (0 to 29 cells/mm^2^) tissue samples (Figure 2). This suggests that the presence of γδ T-cell infiltrates in normal tissues is variable among organs, ranging from medium to high in colon, medium to low in breast tissues, and very low or absent in ovarian and pancreatic tissue sections. We then investigated γδ T-cell infiltration in the corresponding tumor tissues.

### 3.3. γδ T-Cells in Breast Cancer

**Figure 3:**
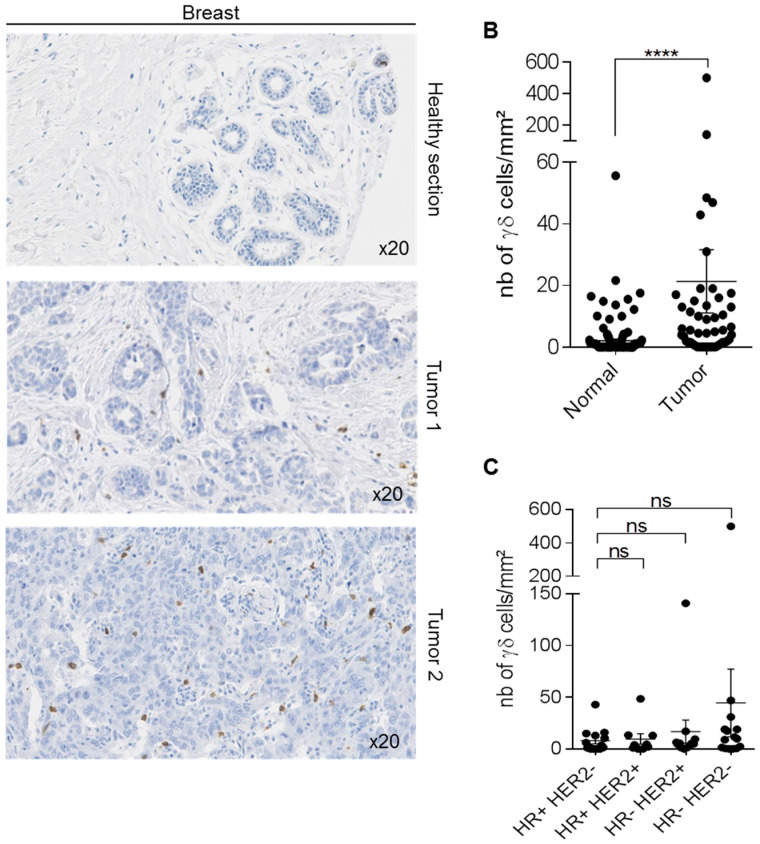
Detection of γδ T-cells in human breast samples. Immunohistochemical detection of γδ T-cells in 50 human breast cancer specimens. (**A**) One representative healthy breast area and two representative breast tumor sections with medium and high γδ T-cell density. (**B**) Scatter plot comparing γδ T-cell density in 141 normal breast samples and 50 breast tumors. (**C**) Scatter plot showing γδ T-cell density in 50 breast cancer samples, according to their hormone receptor (HR) and epidermal growth factor receptor 2 (HER2) status. Data are the mean ± SEM. **** *p* < 0.0001 (Mann–Whitney test for panel B); ns: non-significant (Kruskal–Wallis with Dunn’s multiple comparison test for panel C).

We first compared γδ T distribution in 50 breast cancer samples from patients who did not receive any neo-adjuvant treatment, as well as in 141 normal breast samples, and found that γδ T-cell density was significantly higher in tumors than in healthy breast tissue (*p* < 0.001; Figure 3A,B). However, γδ T-cell density was heterogeneous in breast cancer samples (from 1 to 500 cells/mm^2^) (Figure 3B). We previously showed [22] that γδ T-cell density tended to increase in Scarff–Bloom–Richardson (SBR) grade II–III, compared with SBR grade I breast tumors (*p* = 0.0651, SBRI versus SBRII and III with the Mann–Whitney test). Here, we found that γδ T-cell density tended to be higher in triple-negative breast cancer (TNBC) than in the other breast cancer types (Figure 3C). These observations suggest that the presence of γδ T-cells might be associated with advanced tumors (higher density in SBR III samples), and might be higher in TNBC.

### 3.4. γδ T-Cells in Colorectal Cancer

**Figure 4:**
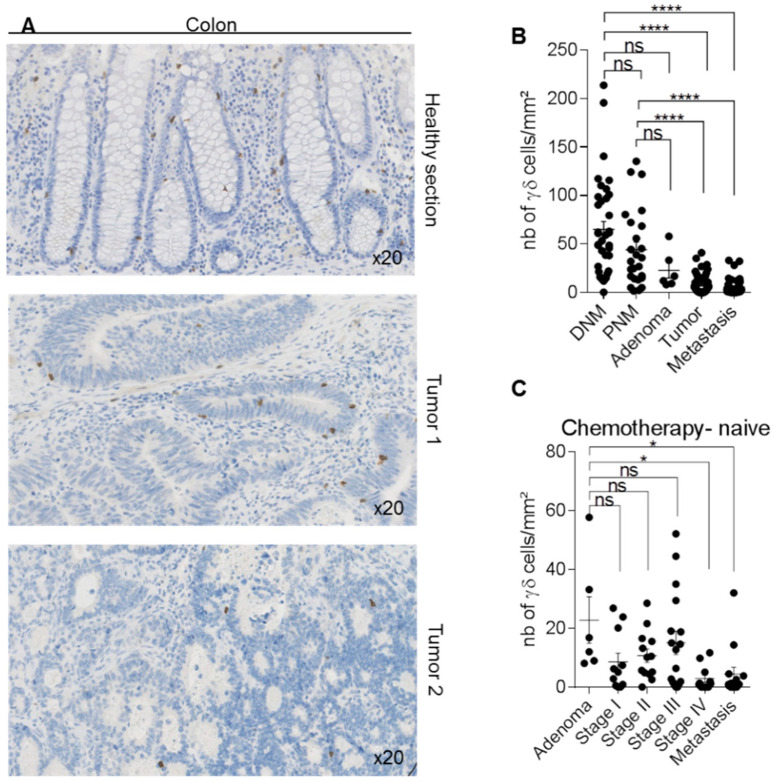
Detection of γδ T-cells in human colon samples. Immunohistochemical detection of γδ T-cells in 112 human colon cancer specimens and normal associated-mucosa (*n* = 62). (**A**) The figure shows one representative normal mucosa section and two representative tumors with medium and low γδ T-cell density. (**B**) Scatter plot comparing γδ T-cells density in 37 distal normal mucosa (DNM), 25 proximal normal mucosa (PNM), 6 adenoma, 58 primary tumor samples, and 48 metastasis/recurrent tumor samples from the two TMAs, described in Materials and Methods. (**C**) Scatter plot showing γδ T-cell density according to the tumor stage in 72 colon cancer samples obtained from chemotherapy-naïve patients. Data are the mean ± SEM. Ns: non-significant; * *p* < 0.05; **** *p* < 0.0001 (Kruskal–Wallis with Dunn’s multiple comparison test).

We then analyzed two TMAs that included, in total, 112 colorectal cancer samples (6 adenomas, 58 adenocarcinomas at all stages, and 48 metastatic tumors) and 62 normal mucosa samples collected near (proximal mucosa) or at a distance (distal mucosa) from the tumor site. In normal mucosa, we detected γδ T-cells in the stroma and the epithelium (Figure 4A). As observed in breast cancer, γδ T-cell density was heterogeneous among tumors (from 0 to 52 cells/mm^2^) (Figure 4A,B). Conversely, γδ T-cell density was significantly lower in primary tumors (*p* < 0.0001) and metastases (*p* < 0.0001) than in normal mucosa (proximal and distal), without any difference between primary and metastatic tumors (Figure 4B). Interestingly, the location of the tumor at the right or left colon have no impact on the γδ T-cell infiltration at the tumor site (Figure S2A). Comparison of γδ T-cell density according to the tumor *BRAF* and *RAS* mutational status showed a decrease, although not significant, of γδ T-cell density in tumors harboring *BRAF* mutations compared with wild type (*WT*) tumors, whereas it was comparable between *WT* and *RAS* mutated samples (Figure S2B). Furthermore, no difference in γδ T-cell density was observed between patients harboring microsatellite instability and patients with stable microsatellites (Figure S2C). To determine whether γδ T-cell density was different among tumor stages, we excluded all samples from patients who received neo-adjuvant treatment. We did not detect any difference in γδ T-cell density in the adenoma or stage I, II, and III samples. Conversely, γδ T-cell density was significantly lower in the stage IV tumors (*p* < 0.05) and metastatic tumors (*p* < 0.05) compared with the adenoma samples (Figure 4C). These data suggest that γδ T-cell infiltration at the tumor site is higher in early-stage colorectal cancers, and lower in late-stage and metastatic tumors.

### 3.5. γδ T-Cells in Pancreatic Cancer

**Figure 5:**
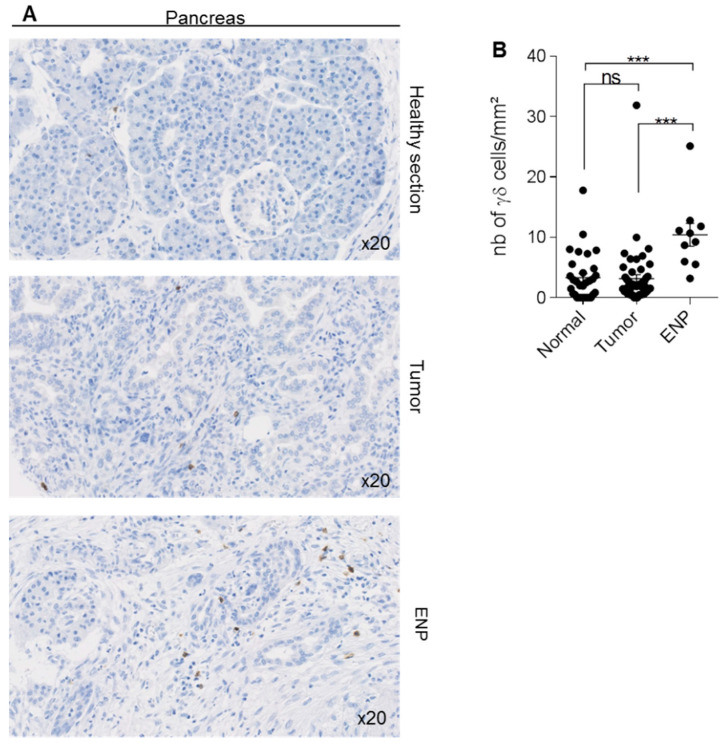
Detection of γδ T-cells in human pancreatic tissue samples. Immunohistochemical detection of γδ T-cells in 50 pancreatic adenocarcinomas, 31 adjacent normal pancreatic sections, and 10 epineoplastic pancreatitis (ENP) samples. (**A**) The figure shows one representative normal pancreas section, one representative tumor section, and one representative ENP section. (**B**) Scatter plot comparing γδ T-cell density in normal, pancreatic cancer, and ENP samples. Data are the mean ± SEM. Ns: non-significant; *** *p* = 0.0001 (Kruskal–Wallis with Dunn’s multiple comparison test).

Analysis of γδ T-cell density in a TMA that included 50 pancreatic adenocarcinomas, 31 adjacent normal pancreatic tissue areas, and 10 epineoplastic pancreatitis samples (i.e., inflamed area close to the neoplastic lesion) showed that the density of γδ T-cells in tumor samples remained low, and was similar to that of normal tissues (3.162 ± 0.6691 cells/mm^2^ vs 3.313 ± 0.7116 cells/mm^2^) (Figure 5). Conversely, γδ T-cell density was significantly increased in the inflamed tissue (10.41 ± 1.899 cells/mm^2^ in epineoplastic pancreatitis sections). 

### 3.6. γδ T-Cells in Ovarian Cancer

**Figure 6:**
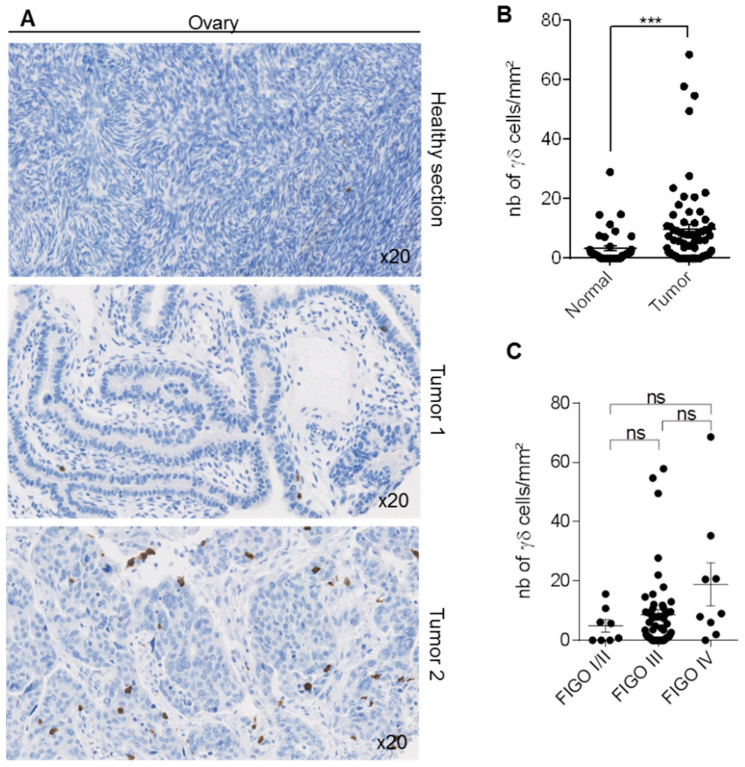
Detection of γδ T-cells in human ovarian samples. Immunohistochemical detection of γδ T-cells in 72 ovarian tumors and 49 paired normal ovarian samples. (**A**) The figure shows one representative normal ovarian tissue and two representative tumors with low and high γδ T-cell density. (**B**) Scatter plot comparing γδ T-cell density in normal and cancer tissue samples. (**C**) Scatter plot showing γδ T-cell expression in the 71 ovarian cancer samples, classified according to the FIGO stage. Data are the mean ± SEM. Ns: non-significant; *** *p* < 0.001 (Mann–Whitney test for panel B, Kruskal–Wallis with Dunn’s multiple comparison test for panel C).

Finally, analysis of γδ T-cell density in a TMA composed of 72 ovarian cancers with normal samples for 49 patients showed that γδ T-cell density was variable among tumors (Figure 6A; from 0 to 64 cells per mm^2^). Nevertheless, γδ T-cell density was significantly higher in tumors than paired healthy tissues (Figure 6B). As in this TMA, more than 25% of tumors were from patients who received neo-adjuvant treatment (Table S4), we wondered if the treatment influenced γδ T-cell density in tumors. The exclusion of all treated patients abrogated the significant difference between normal and tumor tissues, strengthening the hypothesis of an effect of treatment on the γδ T-cell density in ovarian (Figure S3A). In contrast, neo-adjuvant treatments did not influence γδ T-cell infiltration in colon and pancreatic cancer samples (Figure S3B,C). We then compared γδ T-cell density in ovarian tumors classified according to their FIGO stage. We did not find any difference, although γδ T-cell infiltration density tended to be higher in FIGO IV tumors (i.e., advanced cancers) (Figure 6C). 

### 3.7. TNM Classification of Cancers and γδ T-Cell Infiltrates 

**Figure 7:**
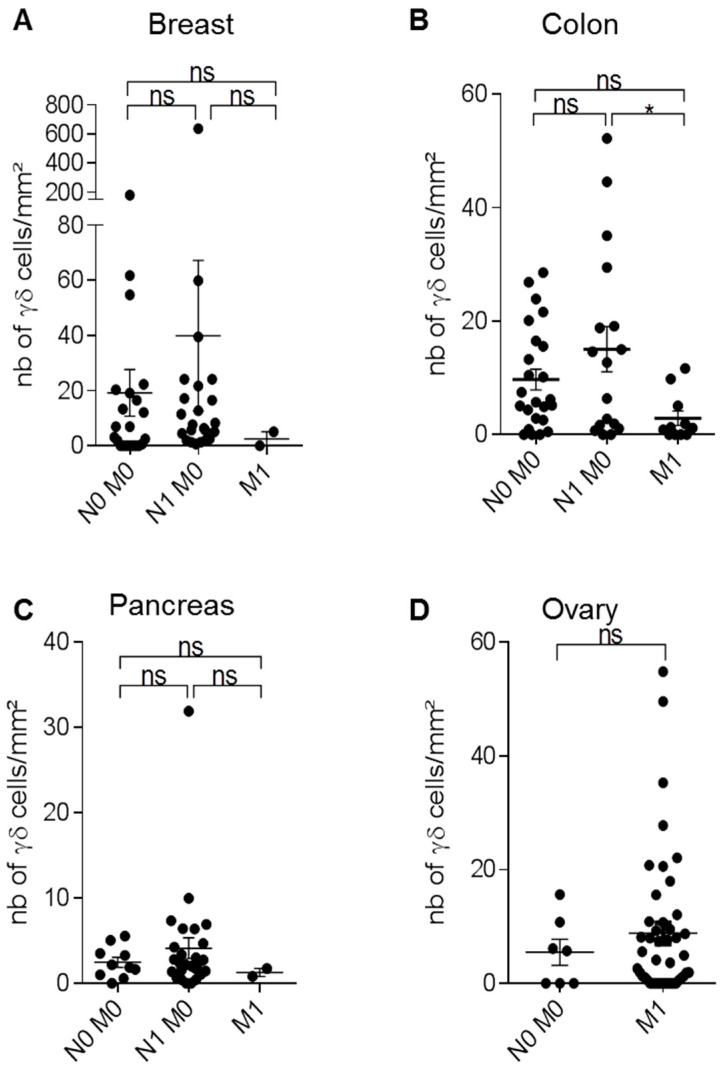
Analysis of γδ T-cell density in function of the tumor, node, and metastasis (TNM) classification. Immunohistochemical detection of γδ T-cells in breast, colon, pancreatic, and ovarian tumors in chemotherapy-naïve patients. Scatter plots comparing γδ T-cell density in N0 M0, N1 M0, and M1 breast (**A**), colon (**B**), pancreatic (**C**), and ovarian (**D**) tumors. Data are the mean ± SEM. Ns: non-significant; * *p* < 0.05 (Kruskal–Wallis with Dunn’s multiple comparison test).

In patients with cancer, prognosis is usually determined following the histopathological analysis of tumor samples resected by surgery. Tumor staging (AJCC/UICC-TNM) is based on tumor size (T), presence of cancer cells in regional and draining lymph nodes (N), and presence or absence of metastasis (M). The TNM classification is used for many solid cancers, and has proven its utility to estimate the patient outcome [23,24,25]. Therefore, after excluding all samples from patients who received neo-adjuvant treatment, we analyzed γδ T-cell density in breast, colorectal, ovarian, and pancreatic cancers, classified according to their TNM status, using the eighth edition of the TNM Classification of Malignant Tumors. In breast, colon, and ovarian cancers, γδ T-cell density was heterogeneous at all stages, while it remained low in all pancreatic cancers (with the exception of one sample) (Figure 7). In colorectal cancer, γδ T-cell density was significantly reduced in M1 tumors compared with N1 M0 tumors. Although not significant, γδ T-cell density was increased in M1 ovarian tumors compared with N0 M0 tumors, and in N1 M0 breast cancers compared with N0 M0 samples. Conversely, γδ T-cell density was very low in M1 breast cancer samples. Altogether, these data indicate that depending on the cancer type, γδ T-cell density was positively or negatively correlated with the TNM stage. 

### 3.8. Association between γδ T-Cell Infiltrates and TILs

The association between tumor-infiltrating lymphocytes and clinical outcome has been well-established in different cancers, and these findings have initiated an increasing interest for immune subpopulations as prognostic markers of tumor behavior and treatment response. In this context, we asked whether γδ T-cell infiltration was correlated or associated with all lymphocyte infiltration (TILs). In the four studied cancers, we observed a significant association between γδ T-cell density and TIL infiltration (*p* = 0.008 for breast cancer and *p* < 0.001 for colon, pancreatic, and ovarian cancers; Table 1A–D, respectively). However, γδ T-cell infiltration was only poorly correlated with TIL infiltration in breast (Spearman’s Rho = 0.497), colon (Spearman’s Rho = 0.426), and ovarian cancer (Spearman’s Rho = 0.457), and not correlated in pancreatic cancer (Spearman’s Rho = 0,290) (Table 1E).

## 4. Discussion

In this study, we validated the use of IHC with the H-41, anti-TCRδ monoclonal antibody to analyze γδ T-cell density in normal and pathological tissues. This antibody was first tested and then recommended by Jungbluth and colleagues as a replacement of another antibody that is no longer available [26]. We first analyzed normal breast, colon, pancreatic and ovarian tissues, and observed that γδ T-cell presence and density varied depending on the organ. Then, we investigated γδ T-cell infiltrates in breast, colorectal, pancreatic, and ovarian tumors, and compared these findings with what was observed in the relevant normal tissues. Similarly to normal tissues, γδ T-cell density was heterogeneous in the cancer types, with medium-to-high γδ T-cell density in breast, colon, and ovarian tumors, and few or no γδ T-cells in pancreatic tumor samples. Moreover, γδ T-cell density was higher in breast and ovarian tumors compared with normal tissues. Conversely, γδ T-cell density was reduced in colorectal cancer compared with healthy samples. Interestingly, in the four cancer types, γδ T-cell infiltration is associated with TIL infiltration when measured as discrete value after comparison with median expression (*p* = 0.008 for breast cancer and *p* < 0.001 for colon, pancreatic, and ovarian cancers; Pearson’s chi-squared test), and classically recommended neo-adjuvant treatments did not impact γδ T-cell infiltration and density. Finally, γδ T-cell density was low in both healthy and pancreas cancer samples, consistent with the fact that pancreatic tumors are considered “cold” tumors (i.e., poorly-infiltrated tumors) [27]. These observations suggest that γδ T-cell presence or recruitment in tissues is context- (healthy or pathological) and organ-dependent, and is associated with global TIL infiltration.

In breast cancer, γδ T-lymphocyte density was highly heterogeneous between tumor samples (Figure 3A-B), but progressively increased from grade I to grade II and III tumors [22]. This suggests, as previously reported by others [28], that in breast cancer, the presence of γδ T-cells is associated with late tumor grade and poor prognosis. Moreover, we found that γδ T-cell density tended to be higher in TNBC than in other breast cancer types (Figure 3C). This difference was not significant, probably due to an insufficient number of samples for each type. TNBCs are very heterogeneous tumors: some are very aggressive with poor prognosis, while others display similar or better prognoses than hormone receptor-positive breast cancers [29]. This suggests that other parameters must intervene in breast cancer prognosis and progression, such as the tumor immune environment. Ye et al showed that regulatory γδ T-cells are present in the breast tumor microenvironment, supporting the idea that their presence in the tumor microenvironment is associated with poor prognosis [30]. Conversely, Hidalgo et al. demonstrated the presence of γδ T-cells in TNBC and suggested an anti-tumor role of this T-cell sub-population [31]. More recently, Wu et al. showed, in a small cohort of patients with TNBC, that the presence of cytotoxic γδ T-cells (specifically Vδ1+ T-cells) is associated with remission and good overall survival, suggesting that in this context, the presence of γδ T-cells is a good prognosis [32]. More studies are needed to determine the role of the different γδ T-cell subpopulations in breast cancer progression, and to analyze their specific role in various breast cancer types.

Prognosis of patients with colon cancer depends on several factors, such as tumor molecular status (microsatellites instability, *BRAF* mutations), localization (right or left side), stage at diagnosis, and density and composition of the tumor immune infiltrate. In recent years, the distinction between right colon cancer (RCC) and left colon cancer (LCC) has been brought into focus, due to their different outcomes, prognoses, and clinical responses to chemotherapy. Notably, the outcomes were superior for patients with left-sided tumors than for those with right-sided tumors [33]. In the present study, we analyzed γδ T-cell density in RCC and LCC and found no difference. Concerning the microsatellite instability status (MSI) that results from impaired DNA mismatch repair (MMR), a meta-analysis pooling 32 studies concluded that MSI was associated with better prognosis when compared to patients with intact MMR [34]. In our study, MSI/MSS status was known for 55 out of 112 patients included in the cohort, and we found that γδ T-cell density was not impacted by the microsatellite status. However, the number of MSI samples were very low (nine MSI versus 46 MSS); thus, examination of a larger cohort might be required to make a definite conclusion.

The *BRAF* mutation is a novel biomarker that is gaining interest due to its association with a worse prognosis when compared to *BRAF* wild-type colon cancer [35,36]. A recent meta-analysis found that colon cancer patients with *BRAF* mutations had worse overall survival (OS) and PFS following anti-EGFR therapies compared to patients with wild-type *BRAF* tumors. Other mutations are also important, in particular for response to treatment. Patients with *Ras* mutations (especially *K-ras*) are unlikely to benefit from anti-EGFR therapy [37]. In the present study, γδ T-cell density tended to be lower (although not significantly) in *BRAF*-mutated samples compared with *WT* and *RAS*-mutated samples (*n* = 69 samples (Figure S2B)). This difference was only significant when the analysis was performed in metastatic samples (data not shown). Our observations are in accordance with several studies showing that *BRAF* mutations are related to poor prognosis in metastatic colon cancer [38], and that disease-free survival is longer in patients with colon cancer harboring abundant γδ T-cell infiltrates [39], suggesting a beneficial anti-tumor role of γδ T-cells.

In agreement, γδ T-cell density was lower in stage IV and metastatic samples compared with adenoma samples. These data correlate with the concept of the Immunoscore, a classification method based on the presence and density of effector cells, especially CD8 T-cells, in the tumor micro-environment. Many studies have shown that patients with colon cancer and low Immunoscores (i.e., low CD3 and cytotoxic CD8 cell infiltration at the tumor site) have poor prognoses, with reduced survival chances. Conversely, patients with high immune infiltrates in the tumor (i.e., a high Immunoscore) have good prognoses [25,40].

A meta-analysis using CIBERSORT showed that an intra-tumoral γδ T-cell signature emerged as one of the most significant, favorable, cancer-wide prognostic populations [41]. Moreover, γδ T-cell and CD8 T-cell signatures were the most highly correlated, suggesting that intra-tumoral γδ T-cell density could be a good prognostic factor. In colon cancer, surgery remains the first line of treatment, and the use of adjuvant therapies depends on the cancer stage. The current protocols recommend adjuvant therapy for stage III and IV colon cancer, while its use is still debated for patients with stage II cancer. Future studies should determine whether the analysis of γδ T-cell density could improve treatment decision-making for patients with non-advanced colon cancer (particularly stage II), and whether γδ T-cell density might become a predictive biomarker for adjuvant therapies.

Pancreatic cancer is a tumor with high malignancy, morbidity, and mortality for several reasons. First, its diagnosis is often very late, when tumors are already at advanced stages. Second, pancreatic cancer displays low immunogenicity, partly due to its low mutation burden, which may explain the lack of tumor-infiltrating effector T-cells [42]. Particularly, the number of CD8 T-cells, which is correlated with good clinical response to immunotherapy, is significantly lower in “non-immunogenic” cancers, such as pancreatic cancer, compared with “immunogenic” cancers, such as melanoma [43]. Also, it has often been reported that effector T-cells are excluded from the pancreatic tumor microenvironment [27,44]. In agreement, we found that γδ T-cell density was low in pancreatic cancer samples, whereas it was much higher in epineoplastic pancreatitis samples, a zone rich in immune cells that can surround pancreatic tumors in certain cases. This finding is in agreement with the exclusion of effector cells from the tumor microenvironment and their re-localization in inflammatory areas [45].

Ovarian cancer is another devastating cancer, because at diagnosis, the tumor is at advanced clinical stages (III/IV) in more than 70% of patients. Moreover, more than 70% of patients will experience tumor relapse, and the five-year overall survival rate is lower than 30% [46]. In our study, γδ T-cell density was higher in tumor specimens (although heterogeneous) compared with healthy tissue samples. Similarly, Chen and collaborators showed after γδ TIL isolation that the Vδ1+ subset, the main population in ovarian cancer, displays reduced cytotoxic activity and inhibits the proliferation of CD4+ T-cells [47]. These authors concluded that γδ T-cells might have critical roles in ovarian cancer progression.

Finally, we investigated γδ T-cell density in tumors classified according to their TNM stage (Figure 7). We observed that γδ T-cell density tended to be lower in advanced stages, particularly the M1 stage, in breast, pancreas, and colon cancers (statistically significant for colon cancer). This suggests that γδ T-cells could be of good prognosis, notably through their anti-tumor functions in these cancers, as already reported [9,11,12,48]. However, for breast cancer, we had only two samples in the M1 stage. That is not enough to definitely determine that there was no difference, or whether the absence of significant difference was due to the low number of samples. Concerning pancreatic cancer, a very low γδ T-cell density in the tumors was observed whatever the TNM stage of the patient. Conversely, γδ T-cell density tended to be higher in M1-stage ovarian cancers, suggesting a pro-tumor function of γδ T-cells in this tumor type (Figure 7), and reinforcing the idea that the presence of γδ T-cells is correlated with poor prognosis in ovarian tumors [49,50]. Probably because of the late diagnosis and the rapid evolution of the disease in ovarian cancer, the analyzed cohort contained few N0 M0 or no N1 M0 samples, and most samples were from patients at the M1 stage. Once again, more samples are required to confirm a significant increase of γδ T-cell density in the advanced stages of ovarian cancer. Overall, these results show that γδ T-cell density is variably associated with the cancer stage or progression, differently that CD8+ T-cells, the presence of which is correlated with good prognosis. This is also in accordance with the conclusions of a microarray data analysis showing a discrepancy between the density of CD8 T-cells and of the Vγ9Vδ2 subset of γδ T-cells in the microenvironment of various tumors [41].

Several recent studies have brought evidence that γδ T-cells are not always associated with good prognosis in some solid cancers. Indeed, besides their anti-tumor functions, γδ T-cells have been associated with pro-tumor functions, either directly through immunosuppression mechanisms or indirectly by favoring the establishment of an immunosuppressive environment through the recruitment of suppressive cells. Specifically, γδ T-cells through interleukin (IL)-17 production display pro-tumor functions in colorectal cancer [50,51,52,53]. On the contrary, in other cancers, IL-17-producing cells can have anti-tumor functions. Ma et al. showed that IL-17-producing γδ T-cells contribute to the chemotherapy-induced anticancer immune response [54]. And Lo Presti et al. demonstrated that the recruitment of γδ T-cells producing either IL-17 or interferon (IFN)-γ depended on the tumor stage [14]. In breast cancer, Peng and colleagues [55] associated γδ T-cell immunosuppressive functions with the induction of dendritic cell senescence and inhibition of T-cell proliferation. However, in this study, no discrimination has been done according to breast cancer subtypes. Conversely, Wu et al. demonstrated the anti-tumor functions of γδ T-cell in TNBC and the good prognostic value of high γδ T-cell density in this type of cancer [32]. Our data shows diversity in the γδ T-cell density between the tumor localization or tissue, corroborating this discrepancy in various studies. These apparent discrepancies could actually reflect the need to better know γδ T-cell density in the different cancer subtypes, and to identify γδ T-cell subsets. Wu et al. showed in TNBC that the main γδ T-cells were δ1 subsets [32], while we had previously reported in a study analyzing breast cancer types that the δ2 subset was also well-represented in the tumors [22]. In pancreatic ductal adenocarcinoma, γδ T-cells inhibit αβ T-cell activation and infiltration via PD-L1 ligation, thereby allowing tumor progression [56]. Overall, these data support the hypothesis that some γδ T-cell subsets can be immunosuppressive and favor tumor progression in selected solid tumor types. Similarly, we recently identified a suppressive γδ T-cell population that expresses CD73, produces IL-10 and adenosine, and is present in breast tumors [22,57]. In this context, to refine the prognostic value of γδ T-cells in the tumor microenvironment, it could be interesting to analyze the ratio of anti- vs. pro-tumor γδ T-cell subsets based on their CD73 expression.

A better knowledge of the immune landscape in tumors is required to identify new predictive and prognostic biomarkers. Whether γδ T-cells (if the discrimination between effector and regulatory subsets is achievable) could be one of these potential new biomarkers remains to be demonstrated.

## Figures and Tables

**Table 1:** Association and correlation analyses between γδ T-cells and tumor-infiltrating lymphocyte (TIL) densities. Association between the γδ T-cell density and TIL density in (**A**) breast cancer (*p* = 00.8), (**B**) colon cancer (*p* < 0.001), (**C**) pancreatic cancer (*p* < 0.001) and (**D**) ovarian cancer (*p* < 0.001) (Pearson’s chi-squared test). High and low densities were defined as values above or below the median values. (**E**) Correlation between the γδ T-cell density and TIL density in all cancers. Spearman’s Rho values are shown in the table. No correlation is observed in pancreatic cancer (Rho < 0.4), while a weak correlation is found in breast, colon and ovarian cancers (Rho < 0.6).

**A**				
**Breast**	**TCRγδ Low**		**TCRγδ High**	
TILs Low	28		15	
TILs High	14		25	
*p* = 0.008 (Pearson Chi2 test).				
**B**				
**Colon**	**TCRγδ Low**		**TCRγδ High**	
TILs Low	69		33	
TILs High	29		62	
*p* < 0.001 (Pearson Chi2 test).				
**C**				
**Pancreas**	**TCRγδ Low**		**TCRγδ High**	
TILs Low	35		19	
TILs High	15		33	
*p* < 0.001 (Pearson Chi2 test).				
**D**				
**Ovary**	**TCRγδ Low**		**TCRγδ High**	
TILs Low	44		22	
TILs High	20		42	
*p* < 0.001 (Pearson Chi2 test).				
**E**				
**Cancer**	**Breast**	**Colon**	**Pancreas**	**Ovary**
**Spearman’s Rho**	0.497	0.426	0.29	0.452

## Supplementary Materials

The following are available online at https://www.mdpi.com/2073-4409/9/6/1537/s1: Figure S1: Validation of the H-41 anti-TCRδ antibody in cell suspensions by immunohistochemistry. Figure S2: Detection of γδ T-cells in human colon samples. Figure S3: γδ T-cell density in colon, pancreas, and ovary tumors from chemotherapy-naïve patients. Table S1: Main clinicopathological characteristics of the cohort of patients with breast cancer. Table S2: Main clinicopathological characteristics of the cohort of patients with colon cancer. Table S3: Main clinicopathological characteristics of the cohort of patients with pancreatic cancer. Table S4: Main clinicopathological characteristics of the cohort of patients with ovarian cancer.
