# Supplementary material for: Diversity of Tumor-Infiltrating, γδ T-Cell Abundance in Solid Cancers
Source: Cells. 2020 Jun 24;9(6):1537. doi: 10.3390/cells9061537 (PMC7348937; doi:10.3390/cells9061537)
Supplement: Supplementary file 1 [file cells-09-01537-s001.pdf]

Appendix A

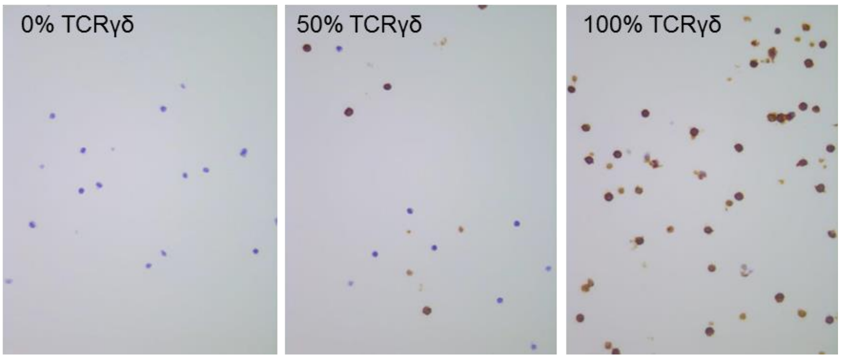

Supplemental figure 1. Validation of the H41 anti-TCRδ antibody in cell suspensions by immunohistochemistry.
γδ T cells were purified from PBMCs of healthy donors by positive immunoselection using a kit from Miltenyi Biotec. PBMCs depleted of γδ T cells were used to prepare cell suspensions that contained different percentages of purified γδ T cells, as indicated. Cell suspensions were embedded in an aqueous gel solution for histological and immunohistochemistry analysis with the H-41 anti-TCRγδ monoclonal antibody, as described in Materials and Methods.

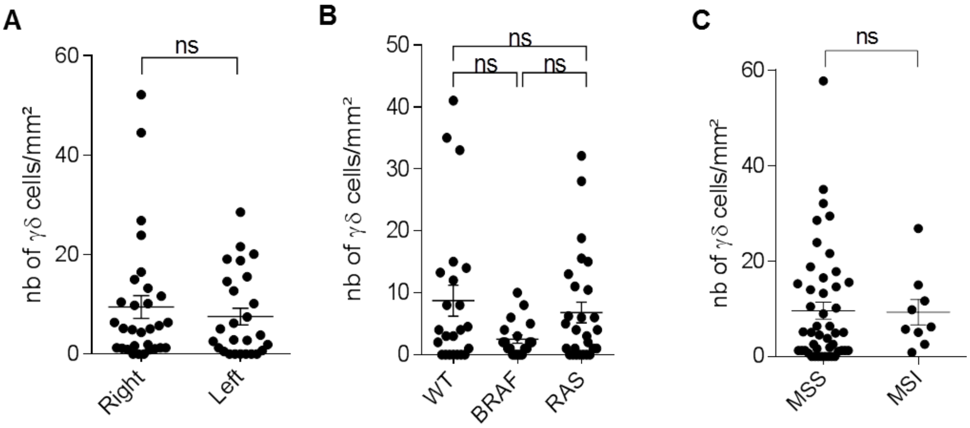

Supplemental figure 2. Detection of γδ T cells in human colon samples. Detection by immunohistochemistry of γδ T cells in human colon cancer. (A) Scatter plot showing γδ T cell density in 56 colon cancer samples depending on the localization (left colon vs right colon). (B) Scatter plot showing γδ T cells density in the 77 colon cancer samples with mutational status information (WT, BRAF and RAS). (C) Scatter plot showing γδ T cells density in 55 colon cancer samples with microsatellite stability status information (mss : stable ; msi : instable). Data are the mean ± SEM. ns, non-significant; *p<0.05; ****p<0.0001 (Kruskal Wallis with Dunn's multiple comparison test).

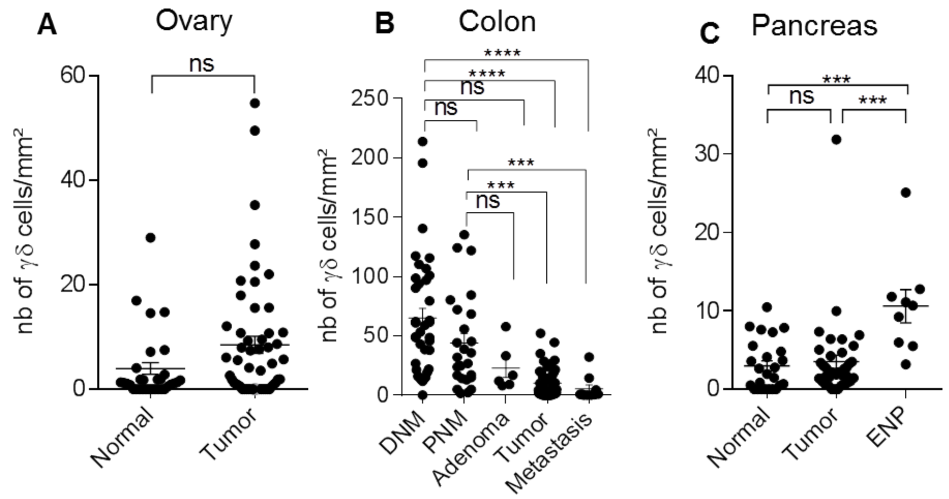

Supplemental figure 3. γδ T cell density in colon, pancreas and ovary tumors from chemotherapy-naive patients.
Detection by immunohistochemistry of γδ T cells in ovarian (A), colon (B) and pancreatic (C) cancer samples from patients who did not receive any neo-adjuvant therapy before tumor surgery. Data are the mean ± SEM. ns, non-significant; * $p<0.05$ (Kruskal Wallis with Dunn's multiple comparison test).

Parameter	Total (n= 50)
Median age at surgery (range)	61.5 (30 - 88)
T stage	
T1	21 (42%)
T2	19 (38%)
T3	6 (12%)
T4	3 (6%)
Missing	1 (2%)
N stage	
N0	22 (44%)
N1	11 (22%)
N2	6 (12%)
N3	8 (16%)
Missing	3 (6%)
M stage	
M0	45 (90%)
M1	2 (4%)
Missing	3 (6%)
Grade	
I	8 (16%)
II	20 (40%)
III	22 (44%)
Breast cancer subgroup	
RH+ HER2-	14 (28%)
RH+ HER2+	9 (18%)
RH- HER2+	12 (24%)
RH- HER2-	15 (30%)
Neoadjuvant treatment	
Yes	0 (0%)
No	50 (100%)

Supplemental table 1. Main clinicopathological characteristics of the cohort of patients with breast cancer

Parameter	Total (103 patients/112 samples*)		
Sex	M		65
	F		38
Median age at surgery [range]		66 [25-91]	
RAS/BRAF mutations			
	RAS mutated		27
	BRAF mutated		19
	RAS/BRAF wt		23
	Undetermined		43
Microsatelitte status			
	MSS		46
	MSI		9
	Undetermined		57
Adenoma		**6**	
Primary tumors		**58**	
	Right colon		30
	Left colon		26
	Missing		2
	Post-CT		6[1]
	Chemo-naive		52
	Stage I		11
	Stage II		13
	Stage III		17
	Stage IV		11
Metastasis		**45**	
	Liver		23
	Peritoneum		17
	Lung		3
	Lymph node		2
	Post-CT		32[2]
	Chemo-naive		13
Local reccurence		**3**	
	Post-CT		2[3]
	Chemo-naive		1

** 5 patients displayed 2 or more lesions*

[1] all patients received FOLFOX neo-adjuvant chemotherapy

[2] Folfox (16); Folfirinox (4); Folfiri-bevacizumab (3); Xeloda (3); Xelox (2); Folfiri (2); Folfiri/Panitumumab (1); Xeliri-bevacizumab (1)

[3] Folfox (1); LV5FU2 (1)

Supplemental table 2. Main clinicopathological characteristics of the cohort of patients with colon cancer

Parameter	Total (n= 50)
Sex	
M	22 (44%)
F	28 (56%)
Median age at surgery (range)	67 (38-89)
Neoadjuvant treatment	
Yes	11[1] (22%)
No	39 (78%)
For chemo-naive samples only (n=39):	
T stage	
T1	5 (13%)
T2	13 (33%)
T3	20 (51%)
T4	1 (3%)
N stage	
N0	12 (31%)
N1	23 (59%)
N2	4 (10%)
M stage	
M0	36 (92%)
M1	2 (5%)
MX	1 (3%)
R status	
R0	34 (87%)
R1	5 (13%)

[1] Folfirinox (6); Gemcitabine (2); Folfirinox + RT/Gemcitabine (1); Folfirinox + RT/Capecitabine (1); RT/Gemcitabine (1)

RT: radiotherapy

Supplemental table 3. Main clinicopathological characteristics of the cohort of patients with pancreatic cancer

Parameter	Total (n= 72)
Median age at surgery (range)	61 (34-82)
Neoadjuvant treatment	
Yes	18[1] (25%)
No	54 (75%)
FIGO stage	
I	4 (6%)
II	4 (6%)
III	54 (75%)
IV	9 (12%)
Missing	1 (1%)
For chemotherapy-naive samples only (n=54):	
T stage	
T1	4 (7%)
T2	5 (9%)
T3	41 (76%)
Missing	4 (7%)
N stage	
N0	18 (33%)
N1	33 (61%)
Missing	3 (6%)
M stage*	
M0	8 (15%)
M1	45 (83%)
Missing	2 (4%)

[1] Carboplatin/paclitaxel (16); carboplatin/paclitaxel/bevacizumab (2)

* Including peritoneal metastases outside the pelvis, retroperitoneal lymphadenopathy and distant metastasis

Supplemental table 4. Main clinicopathological characteristics of the cohort of patients with ovarian cancer
